# Roles of PTH and FGF23 in kidney failure: a focus on nonclassical effects

**DOI:** 10.1007/s10157-023-02336-y

**Published:** 2023-03-28

**Authors:** Hirotaka Komaba

**Affiliations:** 1 Division of Nephrology, Endocrinology and Metabolism, Tokai University School of Medicine, 143 Shimo-Kasuya, Isehara, 259-1193 Japan; 2 Interactive Translational Research Center for Kidney Diseases, Tokai University School of Medicine, Isehara, Japan; 3 The Institute of Medical Sciences, Tokai University, Isehara, Japan

**Keywords:** Chronic kidney disease-mineral and bone disorder, Fibroblast growth factor 23, Kidney failure, Parathyroid hormone, Secondary hyperparathyroidism

## Abstract

Parathyroid hormone (PTH) and fibroblast growth factor 23 (FGF23) each play a central role in the pathogenesis of chronic kidney disease-mineral and bone disorder (CKD-MBD). Both hormones increase as kidney function declines, presumably as a response to maintain normal phosphate balance, but when patients reach kidney failure, PTH and FGF23 fail to exert their phosphaturic effects, leading to hyperphosphatemia and further elevations in PTH and FGF23. In patients with kidney failure, the major target organ for PTH is the bone, but elevated PTH is also associated with mortality presumably through skeletal and nonskeletal mechanisms. Indeed, accumulated evidence suggests improved survival with PTH-lowering therapies, and a more recent study comparing parathyroidectomy and calcimimetic treatment further suggests a notion of “the lower, the better” for PTH control. Emerging data suggest that the link between SHPT and mortality could in part be explained by the action of PTH to induce adipose tissue browning and wasting. In the absence of a functioning kidney, the classical target organ for FGF23 is the parathyroid gland, but FGF23 loses its hormonal effect to suppress PTH secretion owing to the depressed expression of parathyroid Klotho. In this setting, experimental data suggest that FGF23 exerts adverse nontarget effects, but it remains to be confirmed whether FGF23 directly contributes to multiple organ injury in patients with kidney failure and whether targeting FGF23 can improve patient outcomes. Further efforts should be made to determine whether intensive control of SHPT improves clinical outcomes and whether nephrologists should aim at controlling FGF23 levels just as with PTH levels.

## Introduction

Chronic kidney disease (CKD) is a major public health problem, affecting approximately 10 million people in Japan [1] and many people worldwide. CKD is not only a precursor to kidney failure but is also an accelerator of cardiovascular disease and an independent risk factor for death. Indeed, we experienced a substantial number of cardiovascular events and all-cause death during follow-up in the Chronic Kidney Disease Japan Cohort (CKD-JAC), a large-scale prospective cohort of Japanese CKD patients [2]. This is even more critical for individuals with kidney failure receiving dialysis. In the Dialysis Outcomes and Practice Patterns Study (DOPPS), the survival rate of Japanese dialysis patients was quite better than that of patients in other regions, but approximately 10% of our patients died within 3 years of follow-up [3]. Such a huge risk for cardiovascular disease and mortality can only be partly explained by traditional risk factors, and several CKD-specific risk factors have been postulated. Among these, one of the most important and modifiable factors is CKD-mineral and bone disorder (CKD-MBD) [4]. Elevated levels of parathyroid hormone (PTH) and fibroblast growth factor 23 (FGF23) are hallmarks of CKD-MBD and have been implicated in the pathophysiology of adverse outcomes through mechanisms dependent and independent of alterations in mineral and bone metabolism.

This review summarizes the current understanding of the pathophysiological aspects of PTH and FGF23 in CKD and discusses their potential adverse effects with a particular focus on their nonclassical effects.

## Roles of PTH and FGF23 in the pathogenesis of CKD-MBD

One of the early features of CKD-MBD is an increased secretion of PTH, known as secondary hyperparathyroidism (SHPT). Traditionally, the early rise in PTH levels in CKD has been considered to be primarily induced by a progressive decline in 1,25-dihydroxyvitamin D (1,25D) synthesis. The insufficient production of 1,25D could be caused by decreased kidney mass and a functional inhibition of CYP27B1 by hyperphosphatemia in advanced CKD [5], but these metabolic alterations are not commonly seen in early CKD. In this regard, the discovery and characterization of FGF23 have provided an excellent explanation for these observations. Based on accumulated experimental and clinical evidence [6–8], FGF23 is now considered to play a central role in the pathogenesis of CKD-MBD. Circulating FGF23 levels increase during the progression of CKD, presumably as a compensatory response to maintain a normal phosphate balance. This response could prevent the development of hyperphosphatemia, but as a trade-off, it suppresses the biosynthesis of 1,25D, favoring the early development of SHPT [9].

In patients with moderate to advanced stage CKD, normophosphatemia is maintained by the action of FGF23 and PTH to augment phosphaturia, but the progression of CKD together with decreased expression of renal Klotho leads to a reduction in the ability of the kidney to excrete urinary phosphate, leading to the development of overt hyperphosphatemia. This process is accompanied by a progressive reduction in 1,25D levels, which further stimulates PTH secretion. Patients with late-stage CKD thus commonly manifest hyperphosphatemia, decreased 1,25D levels, and SHPT. There is also another, more direct interaction between FGF23 and PTH, which will be described in a later paragraph.

When patients reach kidney failure, circulating levels of PTH and FGF23 increase 5- to 10-fold and 10- to 50-fold above normal, respectively. In this setting, the initiation of hemodialysis leads to progressive reductions in serum phosphorus, PTH, and FGF23 levels, with dialysis-related fluctuations [10]. The magnitude of the FGF23 reductions was strongly associated with concomitant changes in serum phosphorus levels, supporting the view that phosphorus is a strong inducer of FGF23 production. Interestingly, there was also a slight increase in 1,25D levels after initiation of dialysis, suggesting that even in patients with kidney failure, CYP27B1 activity is in part functionally inhibited by FGF23.

## Clinical outcomes related to SHPT

Among patients with kidney failure, bone is recognized as the principal target of PTH. The most common skeletal manifestation of severe SHPT is a high-turnover bone disease (osteitis fibrosa), characterized by excessive rates of bone resorption and formation, progressive bone loss, and increased bone fragility. The association between PTH levels and fracture risk has not been consistent among studies, but the international DOPPS study demonstrated that intact PTH levels above 900 pg/ml are independently associated with an elevated risk for a new fracture [11].

Importantly, the consequence of SHPT is not limited to bone disease. It is also associated with the risk of mortality. This association has been demonstrated in many cohort studies from different regions of the world, including Japan [12–14]. The causality of these associations may be supported by the observations on PTH-lowering treatment. In the Evaluation of Cinacalcet Hydrochloride Therapy to Lower Cardiovascular Events trial, cinacalcet did not significantly reduce the risk of death, but analyses adjusted for baseline covariates or accounting for study-drug exposure showed a significant effect [15]. We and others also reported significant associations between parathyroidectomy (PTx) and better survival [16–18]. Although these data are from observational studies that cannot prove causality, the survival benefit associated with PTx is independent of potential confounders and is consistent across different regions, suggesting a strong beneficial effect of PTx on clinical outcomes [19].

An important question for the treatment of SHPT is how strictly we should control PTH. Given the difference in the magnitude of the PTH-lowering effect, a comparison of long-term outcomes with PTx and cinacalcet may provide a clue to this question. To this end, we analyzed data from the Japanese Society for Dialysis Therapy Renal Data Registry, a nationwide database of dialysis patients in Japan [20], and compared patients who underwent PTx and those who started treatment with cinacalcet after propensity score matching at a 1:3 ratio. As expected, PTx resulted in greater reductions in intact PTH, calcium, and phosphorus levels than cinacalcet. During the 6-year follow-up period, 22.5% in the PTx group and 27.4% in the cinacalcet group died, translating to a hazard ratio of 0.78 (95% CI 0.67–0.91, *P* = 0.002). Of note, when we stratified patients undergoing PTx into tertiles based on their postoperative PTH and repeated propensity score analysis, the survival benefit of PTx was most evident in the lowest postoperative PTH tertile (< 35 pg/mL), further supporting the notion of “the lower, the better” for the treatment of SHPT [21]. There is concern that the oversuppression of PTH after PTx can lead to adynamic bone disease, which might adversely affect bone strength. However, we did not observe a difference in the rates of hip fracture between the PTx and cinacalcet groups, which would argue against the current belief that low PTH should be avoided for maintaining bone strength. Currently, the target ranges for PTH levels in dialysis patients are largely dependent on observational studies and have been a matter of debate [4, 22]. In Japan, the national guideline suggests maintaining intact PTH levels in the range of 60 to 240 pg/mL [22], which is quite lower than the range suggested in the international guideline (approximately 130 to 585 pg/mL) [4]. If intensive control of SHPT improves clinical outcomes, the lower PTH target may in part explain the better survival in Japanese dialysis patients as compared with those in other regions [23]. Further clinical studies are needed to identify the optimal PTH target in patients undergoing dialysis.

## Potential mechanisms linking SHPT and poor outcomes in kidney failure

Another important aspect regarding SHPT pertains to the mechanisms through which elevated PTH could lead to poor clinical outcomes. One possible pathway is fracture events because high PTH levels are associated with fracture risk [11], and these events lead to a marked increase in subsequent mortality [24]. However, the rates of fractures requiring hospitalization are approximately 6-fold lower than that of mortality in the DOPPS [24], indicating that fracture events could explain at most only a small fraction of the increased mortality with elevated PTH. Another possibility is vascular calcification. However, PTH may not directly induce arterial calcification [25], and the associations between PTH and mortality have been independent of serum calcium and phosphorus [16–18], which are potent inducers of calcification. Collectively, the association between SHPT and increased mortality cannot fully be explained by bone disease or vascular calcification.

In this context, one of the other possible mechanisms linking high PTH and poor outcomes is wasting, characterized by increased energy expenditure. In a previous clinical study of hemodialysis patients, elevated PTH was shown to be an independent determinant of increased energy expenditure as measured by indirect calorimetry [26]. Furthermore, the increased energy expenditure decreased significantly in all patients 6 months after PTx. As a potential explanation for these observations, a recent breakthrough revealed a key role of PTH in the pathogenesis of wasting in kidney failure [27]. Prior to the publication of this paper, Spiegelman's group discovered that PTH-related protein (PTHrP), a tumor-derived protein that causes hypercalcemia of malignancy, directly acts on white adipocytes to induce a phenotypic switch to brown adipocytes (a phenomenon termed adipose tissue browning) and thereby triggers energy wasting in fat tissues, leading to cachexia [28]. We had the opportunity to collaborate with this group and were able to show that in a manner analogous to the role of PTHrP in malignancy, PTH also induces adipose tissue browning and causes cachexia in kidney failure. Specifically, we generated mice with fat cell-specific deletion of the PTH/PTHrP receptor and demonstrated that these mice were resistant to adipose browning and wasting induced by 5/6 nephrectomy [27].

These data reveal a direct role of PTH in adipose tissue browning and wasting in kidney failure, but it was not known whether uncontrolled SHPT leads to weight loss, a hallmark of wasting. To address this, we analyzed data from the international DOPPS and found a strong linear correlation between baseline PTH levels and weight loss during the subsequent 12 months [29]. This relationship was sustained after adjustment for numerous covariates and was robust regardless of whether patients were hospitalized. Furthermore, the association between PTH and weight loss partly mediated the higher risk of mortality associated with elevated PTH levels. These findings support our experimental evidence for the role of PTH in wasting [27] and suggest that this pathway may be a mediator between elevated PTH levels and mortality in dialysis patients.

Studies also suggest that SHPT is involved in the pathogenesis of left ventricular hypertrophy, renal anemia, and immune dysfunction, presumably through the direct action of PTH on each tissue. Supporting these possibilities, clinical studies reported an improvement in these comorbidities after PTx or calcimimetic treatment. A more detailed description of these effects is provided elsewhere [30–32].

The last possible pathway linking SHPT and mortality may be one through increased circulating FGF23 levels. PTH is known as one of the major drivers of FGF23 secretion in osteocytes [33], and in line with this, we and others have shown that both calcimimetics and PTx substantially lower circulating FGF23 levels as well as PTH levels in dialysis patients with SHPT [34, 35]. As detailed below, experimental studies suggest a direct link between FGF23 and multiple organ injury. If so, it could be hypothesized that SHPT leads to poor outcomes through stimulation of FGF23 production. However, it remains to be determined whether FGF23 directly contributes to adverse outcomes in CKD patients, and further work is needed to confirm this possibility.

## Potential off-target effects of FGF23 in kidney failure

In patients with kidney failure, FGF23 loses its ability to stimulate urinary phosphate excretion and inhibit 1,25D production. In this setting, the principal target organ for FGF23 would be the parathyroid gland, in which FGF23 directly inhibits PTH secretion [36]. However, in most patients undergoing dialysis, PTH remains elevated despite extremely high FGF23 levels. To investigate the mechanism for such parathyroid resistance to FGF23, we obtained parathyroid tissues from patients undergoing PTx and found that the expression of Klotho and its coreceptor FGF receptor 1 (FGFR1) is substantially depressed, particularly in nodular hyperplasia, an advanced form of parathyroid hyperplasia [37]. Experimental studies have also reported that in models of CKD, FGF23 fails to suppress PTH secretion, presumably owing to decreased expression of the Klotho-FGFR complex [38, 39]. Of note, a recent mouse genetic study showed that parathyroid-specific deletion of Klotho does not eliminate the PTH-lowering effect of FGF23, but simultaneous deletion of Klotho and calcium-sensing receptors leads to increased PTH production and accelerated parathyroid hyperplasia compared to calcium-sensing receptor deletion alone [40]. This finding suggests a key role of parathyroid Klotho in the regulation of PTH secretion, especially when removing the regulation by calcium-sensing receptors, and supports the concept that depressed parathyroid expression of Klotho and FGFR1 could lead to parathyroid resistance to FGF23 and thereby contribute to PTH hypersecretion and SHPT progression [9].

Another possible target for FGF23 is bone because osteoblasts and osteocytes also express a low amount of Klotho [41]. Although the role of Klotho in bone cells is still not completely understood, recent investigations suggest that FGF23 directly acts on osteocytes by binding to the Klotho-FGFR complex and thereby regulates its own production and bone formation [42, 43]. However, these effects may be offset in kidney failure because the expression of Klotho in bone cells is further attenuated in this setting. The pathophysiological significance of the decreased expression of bone Klotho remains to be determined, but it is possible that it might lead to skeletal resistance to FGF23, weakening the inhibitory effect of FGF23 on bone formation. If so, this may lead to the attenuation of bone loss that is associated with renal osteodystrophy [44]. This scenario is currently hypothetical but may explain the lack of clinical association between serum FGF23 levels and bone mineral density in dialyzed patients [45].

While FGF23 is unlikely to exert its hormonal, Klotho-dependent effects on classical target organs in kidney failure, there is a possibility that FGF23 exerts nonclassical effects in a Klotho-independent manner [46]. Experimental studies suggest that FGF23 has several pathogenic off-target effects, among which the most extensively studied is the effect of inducing left ventricular hypertrophy through the activation of FGFR4 [47, 48]. This finding may well explain the clinical observation of the strong association between elevated FGF23 and mortality [49–53].

However, several lines of evidence challenge this view. The first argument is an absence of the exposure–response relationship in the association between FGF23 and mortality. If we combine the results of previous cohort studies across the spectrum of CKD [49–53], it seems that the association between FGF23 and mortality persists during the predialysis period, starts to be progressively attenuated after initiation of dialysis, almost disappears during long-term dialysis, and then resurges after kidney transplantation. This changing pattern of the FGF23-associated mortality risk is similar to a mirror image of that of circulating FGF23 levels, incompatible with a causal exposure–response relationship. The second is the finding that in most animal models and patients with disorders of primary FGF23 excess, such as X-linked hypophosphatemia, there was no cardiac hypertrophy [54, 55]. One might argue that FGF23 can exert its most potent effects on the heart only in the presence of other contributing factors, such as those seen in CKD. However, this possibility seems unlikely because, as mentioned above, the association of FGF23 with adverse outcomes was not particularly pronounced in patients with advanced CKD. Finally, it should be noted that cardiomyocytes have the potential to produce FGF23. Two independent research groups have demonstrated a profound increase in cardiac expression of FGF23 in animal models of myocardial infarction and cardiac hypertrophy [56, 57]. In keeping with the experimental observations, patients with cardiogenic shock show a tremendous increase in plasma FGF23 levels [58], supporting the view that cardiac injury induces rather than follows the elevation of FGF23 levels. Thus, the relationship between FGF23 and cardiac disease is fairly complex, and it would seem simplistic to attribute the observed associations between FGF23 and mortality to direct cardiac actions of FGF23 [59].

In addition to the potential to induce cardiac hypertrophy, experimental data suggest multiple toxic effects of FGF23, including the induction of inflammation [60], immune dysfunction [61], and anemia [62]. However, even less is known about the clinical impact and relevance of these off-target effects.

## Conclusions

**Fig. 1:**
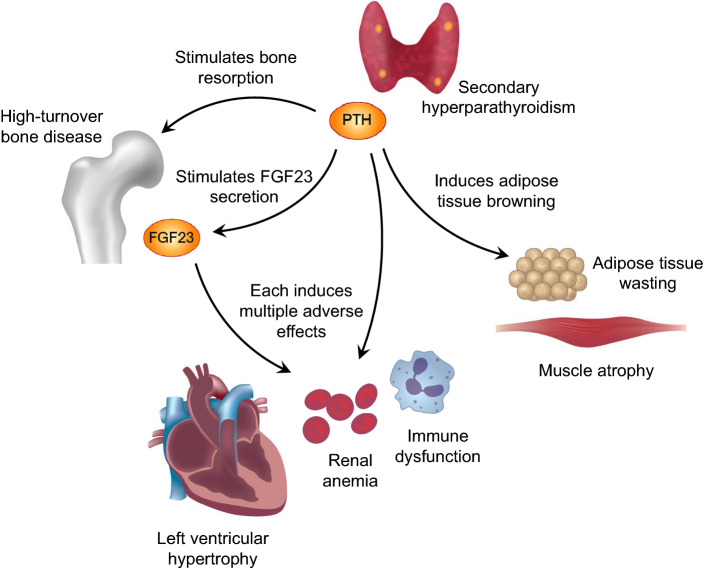
Schematic representation of the adverse effects of parathyroid hormone (PTH) and fibroblast growth factor 23 (FGF23) in kidney failure. Elevated PTH not only causes high-turnover bone disease but also acts on adipose tissue to induce wasting and muscle atrophy. Elevated PTH may also contribute to the pathological development of left ventricular hypertrophy, renal anemia, and immune dysfunction. Part of these effects may be mediated by the effect of PTH to stimulate FGF23 production in the bone

Elevated levels of PTH and FGF23 play a central role in the pathogenesis of CKD-MBD and are also implicated in multiple adverse events in kidney failure, as summarized in Fig. 1. Accumulated evidence suggests that effective treatment of SHPT may not only lead to fracture prevention but also provide a survival advantage, which might in part be mediated by attenuation of PTH-induced wasting. Furthermore, our recent comparison of PTx and cinacalcet treatment suggests a notion of “the lower, the better” for PTH-lowering therapy, which supports the need for future research to test whether intensive control of SHPT improves clinical outcomes. Experimental evidence has also accumulated on the adverse effects of FGF23, but much less is known about whether FGF23 directly contributes to multiple organ injury and whether targeting FGF23 can improve patient outcomes. Thus, more efforts should be made to determine whether nephrologists should measure FGF23 in clinical practice and aim at controlling it just as with PTH.

## Data Availability

Not applicable.
